# Phenotypic features of a microdeletion in chromosome band 20p13: A case report and review of the literature

**DOI:** 10.1002/mgg3.739

**Published:** 2019-05-13

**Authors:** Hung‐Hsiang Fang, Shih‐Yao Liu, Ying‐Fu Wang, Che‐Ming Chiang, Chiung‐Chen Liu, Chien‐Ming Lin

**Affiliations:** ^1^ Department of Pediatrics, Tri‐Service General Hospital National Defense Medical Center Taipei Taiwan; ^2^ Department of Pediatrics National Taiwan University Hospital and College of Medicine, National Taiwan University Taipei Taiwan; ^3^ Department of Radiation Oncology, Tri‐Service General Hospital National Defense Medical Center Taipei Taiwan; ^4^ Graduate Institute of Medical Sciences National Defense Medical Center Taipei Taiwan

**Keywords:** 20p13, array‐comparative genomic hybridization, developmental delays, microdeletion

## Abstract

**Background:**

20p13 microdeletion syndrome has been reported to be associated with developmental delays, intellectual disability, epilepsy, and unspecific dysmorphic characteristics. However, only a few cases of 20p13 microdeletion have been described, and therefore its typical features and precise pathogenesis remain elusive.

**Methods and Results:**

In this article, we report the case of a 9‐month‐old infant who presented with a large fontanelle, facial dysmorphism, and failure to thrive. Array‐comparative genomic hybridization (aCGH) analysis confirmed a 2.01‐Mb microdeletion in chromosome band 20p13 that involved *SOX12* and *NRSN2*, both of which are considered paramount causative genes in patients with 20p13 microdeletion. To elucidate the typical features of 20p13 microdeletion, we further reviewed these previously reported cases and found that motor delay (90%) was the most common manifestation, followed by language delay (60%), abnormal digits (60%), mental retardation (50%), large fontanelle (50%), electroencephalography abnormalities (50%), and seizure (40%).

**Conclusion:**

This report highlights the potential of aCGH as a practical and powerful tool with which to detect submicroscopic chromosomal abnormalities in individuals presenting with a wide spectrum of phenotypes, ranging from facial dysmorphism to failure to thrive. Additionally, the literature review casts new light on the clinical features of 20p13 microdeletion.

## 1 INTRODUCTION

Microdeletion syndrome is a genetic disorder characterized by a small (<5 megabases [Mb]) chromosomal deletion that spans several genes but cannot be detected using conventional cytogenetic methods or high‐resolution karyotyping. Microdeletion syndrome has been associated with intellectual disability, multiple congenital anomalies, and autism spectrum disorders (Lupski & Stankiewicz, 2005). Therefore, a high index of suspicion is needed to ensure an early diagnosis and provide appropriate management.

Previously, a microdeletion of the 20p12.2 locus involving *JAG1* (OMIM#601920) was found to contribute to the well‐known Alagille syndrome (Saleh, Kamath, & Chitayat, 2016). However, the typical presentations and pathogenesis of its near region in terms of 20p13 microdeletion remain elusive. Although a few reports of 20p13 microdeletion have described developmental delays, mild to moderate intellectual disability, seizure disorders, and dysmorphic features (An et al., 2013; McGill et al., 2010; Moutton et al., 2012; Sebat et al., 2007), the manifestations of this genetic event remain under‐reported because variations in deletion size have led to diverse genotype–phenotype associations. In the absence of specific presentations and consistent genetic defects, individuals affected by 20p13 microdeletion may be at risk of delayed clinical diagnosis.

Here, we report the case of a 9‐month‐old female infant who presented with a large fontanelle, facial dysmorphism, and failure to thrive. Array‐comparative genomic hybridization (aCGH) analysis identified her as a carrier of a 20p13 microdeletion of up to 2.01 Mb in size [chr20:g.(60747_2073671)del]. This microdeletion involved *SOX12* (OMIM#601947) and *NRSN2* (OMIM#610666), which were previously reported to induce developmental delays in patients with 20p13 microdeletions (An et al., 2013). This case underscores the potential of aCGH as a useful method for elucidating as‐yet unidentified cases involving dysmorphic features and growth disorders and thus ensuring early diagnosis and appropriate therapy.

## 2 CASE REPORT

**Figure 1:**
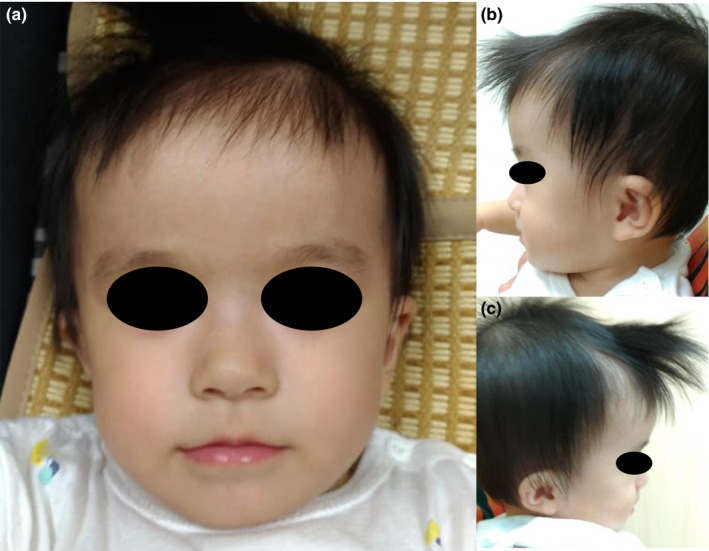
Image of facial dysmorphism in a 9‐month‐old female infant with 20p13 microdeletion syndrome. Note the flat philtrum, thin upper lip, hypertelorism (a), prominent forehead (b), and bilateral low‐set ears (c)

A female Taiwanese infant was referred to an outpatient endocrine clinic for the evaluation of a wide anterior fontanelle and failure to thrive. She was the first child born to healthy and nonconsanguineous parents. A review of the prenatal history revealed a normal three‐vessel umbilical cord and normohydramnios. The subject was born to a 34‐year‐old mother by spontaneous vaginal delivery at a gestational age of 39 weeks. At birth, her body weight, body length, and head circumference were 3,300 g (50–75th percentile), 48 cm (10–25th percentile), and 33.5 cm (50th percentile), respectively. A postnatal physical examination revealed a large fontanelle (4 × 4 fingerbreadth), prominent forehead, hypertelorism, flat philtrum, thin upper lip, low set ears (Figure 1), and bilateral postaxial polydactyly. The remaining physical examination findings were unremarkable. She underwent reconstruction of bilateral polydactyly at 2 days of age.

The subject was brought to our pediatric endocrine clinic at the age of 7 months because of poor weight gain and a persistently wide fontanelle. At that time, her body weight, body length, and head circumference were 5,800 g (<third percentile), 62 cm (<third percentile), and 42.5 cm (25–50th percentile), respectively. She was unable to roll over or sit unassisted, and a backward head position was observed when she was pulled up from her bed. Her short stature and failure to thrive warranted an analysis of thyroid function, which revealed a free T4 level of 1.10 ng/dl (normal: 0.89–1.78 ng/dl), T4 level of 5.49 µg/dl (5.38–12.40 µg/dl), and thyroid‐stimulating hormone level of 1.90 µIU/ml (0.25–5.00 µIU/ml). Venous blood gas analysis yielded a pH of 7.405 (7.32–7.45), PCO_2_ of 28.9 mmHg (41–51 mmHg), PO_2_ of 63.8 mmHg (20–49 mmHg), HCO_3_ of 17.7 mmol/L (24–28 mmol/L), and base excess of −5.3 mmol/L ([−10]–[−2] mmol/L). Metabolic profile analysis revealed an NH_3_ level of 110 µg/dl (68–136 µg/dl) and a lactate level of 2.1 mmol/L (0.6–3.2 mmol/L). Urine organic acid profile and plasma ammonic acid studies yielded negative results.

**Figure 2:**
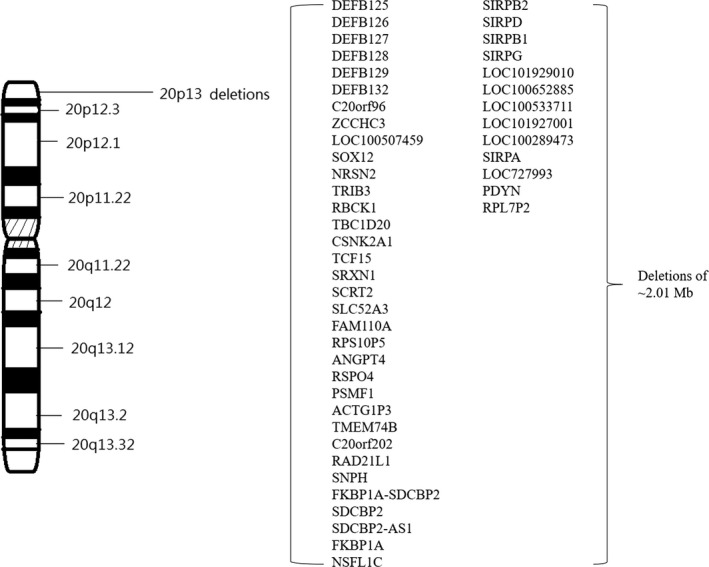
Schematic view of chromosome 20. A zoom of the 20p13 region shows the deleted genes. The observed microdeletion in 20p13 was up to 2.01 Mb in size (genomic location: chr20:60,747‐2,073,671)

Chromosome analysis revealed no chromosomal aberrations, despite the observed facial dysmorphism. In addition, brain magnetic resonance imaging showed no prominent Virchow–Robin spaces, abnormal basal ganglia, or cerebral atrophy. Accordingly, oligonucleotide aCGH analysis was conducted, which detected a microdeletion of up to 2.01 Mb at chromosome 20p13 [chr20:g.(60747_2073671)del]. This microdeleted chromosomal region contains the following OMIM genes: *SOX12* (OMIM#601947), *NRSN2* (OMIM#610666), *TRIB3* (OMIM#607898), *RBCK1* (OMIM#610924), *TBC1D20* (OMIM#611663), *CSNK2A1* (OMIM#115440), *TCF15* (OMIM#601010), *SLC52A3* (OMIM#613350), *FAM110A* (OMIM#611393), *ANGPT4* (OMIM#603705), *RSPO4* (OMIM#610573), *SNPH* (OMIM#604942), *FKBP1A* (OMIM#186945), *SIRPG* (OMIM#605466), *PDYN* (OMIM#131340), ect (Figure 2). The above microdeletion was not detected in her parents, as it was considered de novo.

Electroencephalography (EEG) was performed to survey potential associated clinical defects. This exam revealed a bilateral occipital background with high‐voltage synchronous spikes and waves in F3 and F4. Rapid bilateral frontal poly spikes and C3 spikes were also detected. An abdominal sonography and echocardiogram showed no significant abnormalities. Although a normal fundus was observed by an ophthalmologist, bilateral sensorineural hearing loss was confirmed by an otolaryngologist.

## 3 DISCUSSION

We report the case of a female infant who presented with a large fontanelle, facial dysmorphism, and failure to thrive, which were attributed to a subtelomeric deletion of 20p13. This deletion included *SOX12* and *NRSN2*, both of which have been identified as pivotal genes associated with developmental delays (An et al., 2013). Notably, the pure microdeletion of 20p13 without other genetic defects is rare, and such events involve a wide range of chromosomal deletion sizes. Therefore, it is difficult to establish a genotype–phenotype correlation in affected patients (Jezela‐Stanek, Kucharczyk, Pelc, Gutkowska, & Krajewska‐Walasek, 2013). Although 20p13 deletions may be associated with developmental delays in motor and speech, mental retardation and epilepsy at various levels of severity, and facial dysmorphism (Moutton et al., 2012), the typical characteristics of such cases remain enigmatic, leading to diagnostic delays. Interestingly, previous studies showed that patients who simultaneously harbored a duplication and subtelomeric deletion of 20p exhibited facial dysmorphism, psychomotor retardation, delayed development, and speech difficulties (Leclercq et al., 2009; Trachoo, Assanatham, Jinawath, & Nongnuch, 2013). However, the precise mechanisms underlying duplications and/or deletions of the 20p13 chromosome band remain unclear and require further study.

To date, only a few cases of 20p13 microdeletion have been reported, and most involved de novo events like our patient (An et al., 2013). To elucidate the typical features of 20p13 microdeletion, we further reviewed these previously reported cases and found that motor delay (90%) was the most common manifestation, followed by language delay (60%), abnormal digits (60%), mental retardation (50%), large fontanelle (50%), EEG abnormalities (50%), and seizure (40%) (Table 1) (An et al., 2013; Baker et al., 2002; Jezela‐Stanek et al., 2013; McGill et al., 2010; Moutton et al., 2012). Last but not least, facial dysmorphism was identified as a crucial presentation even though its occurrence varied from 30% to 70% and depended on the involved facial sites. As all reported patients with 20p13 microdeletion exhibited at least one facial abnormality, we emphasize that microdeletion syndrome should be considered in any patient who presents with facial dysmorphism to facilitate an early diagnosis and appropriate management. To be closer to the real‐world clinical practice, 52 additional cases exhibited pathogenic condition were also reviewed through ClinVar database. Similar to Table 1, the most common phenotype of these informally reported cases was developmental delays (13 cases, 25%) followed by facial dysmorphism (five cases, 10%). Interestingly, we found that most of the affected patients with developmental delays were caused by the defects of multiple genes which were usually associated with *TBC1D20, SOX12,* and *NRSN2* genes. Therefore, further functional study is required to elucidate the mechanisms behind those causative genes and developmental process.

**Table 1:** Comparison of phenotypic features of the patients with a 20p13 subtelomeric microdeletion

	Yu An et al.				McGill et al.		Baker et al.	Moutton et al.	Jezela‐Stanek et al.	Our case	Affected/Total case (%)
	Case 1	Case 2	Case 3	Case 4	Case 1	Case 2	Case 1	Case 1	Case 1		
Size of deletion	1.4 Mb	1.1 Mb	799 kb	274 kb	1.7 Mb	1.2 Mb	NA	2.08 Mb	1.15 Mb	2.01 Mb	
Gender	Male	Male	Female	Male	Female	Female	Male	Female	Female	Female	
Diagnostic age	16 years 9 months	11 years 6 months	3 years 4 months	16 years	11 months	15 years	10 years	19 years	9 years	9months	
Large fontanelles	−	+	−	NA	+	+	NA	−	+	+	5/10 (50%)
Forehead	Normal	Frontal bossing	Frontal bossing	Normal	Normal	NA	NA	Normal	Normal	Frontal bossing	3/10 (30%)
Head	Normal	Macrocephaly	Macrocephaly	Normal	Normal	Microcephaly	NA	Microcephaly	Normal	Normal	4/10 (40%)
Hearing	NA	NA	Normal	Normal	NA	NA	NA	NA	NA	Bilateral sensorineural hearing loss	1/10 (10%)
Vision	NA	NA	NA	Normal	NA	Poor visual acuity	NA	Peripheral retinopathy	Normal	Normal	2/10 (20%)
Facial feature											
Prominent nasal root and ridge	−	−	A short nasal root with broad tip and anteverted nares	−	−	+	NA	+	−	−	3/10 (30%)
Eyes	Narrow palpebral fissures	Deep‐set eyes	Downslanting palpebral fissures, slightly deep set eyes	Slightly hypertelorism	Microcorneas, marked edema of the lids, reversed epicanthal, synophrys, telecanthus	NA	NA	Normal	Normal	Hypertelorism	6/10 (60%)
Ears	Hypoplastic helix	−	−	−	Thicked, posteriorly rotated	Low‐set and posteriorly rotated ears with thickened overfolded helices	NA	Low‐set, dysplastic, thick overfolded helices	−	Low‐set ears	5/10 (50%)
Flat philtrum	NA	Everted	NA	NA	+	−	NA	−	+	+	4/10 (40%)
		philtrum									
Thin upper lip	Normal	Inverted upper lip	Normal	Bowed upper lip	+	+	NA	+	+	+	7/10 (70%)
High palate	+	−	−	−	−	+	NA	+	+	−	4/10 (40%)
Others											
Hypoplastic nails	+	−	NA	−	−	+	NA	Broad finger tips	+	−	4/10 (40%)
Digits	Normal	Syndactyly	NA	long toes	NA	Short, broad thumbs	NA	Brachydactyly	discrete fifth finger clinodactyly	Polydactyly	6/10 (60%)
Seizures	−	−	NA	−	Neonatal seizure	Generalized seizure	Generalized seizure	Absence‐epilepsy	−	−	4/10 (40%)
EEG abnormalities	+	+	NA	−	NA	+	NA	+	NA	+	5/10 (50%)
Weight deficiency	−	−	−	−	−	+	NA	−	+	Growth retardation	3/10 (30%)
Neonatal complication	Jaundice	−	Gastroesophageal reflux	NA	Respiratory stress	Mild jaundice	NA	Jaundice	NA	NA	5/10 (50%)
Developmental delay											
Motor delay	+	+	+	+	+	+	NA	+	+	+	9/10 (90%)
Language delay	+	+	+	+	NA	+	NA	+	−	na	6/10 (60%)
Mental retardation	−	Mild	−	NA	NA	Mild	Moderate	Moderate	+	na	5/10 (50%)

Note

NA, no available; +, present; −, absent; na, no assessment.

Among the affected cases on ClinVar database, 16 patients with 20p13 microdeletion exhibited *TBC1D20* gene deletion (16/52, 30%), and 10 out of them (63%) were reported to have developmental delays, suggesting the importance of *TBC1D20* gene for neurological development. Furthermore, Warburg Micro syndrome‐4 (WARBM4) (OMIM#615663) caused by homozygous mutation of the *TBC1D20* gene has been associated with corpus callosum hypoplasia and severe mental retardation (Liegel et al., 2013; Martsolf, Hunter, & Haworth, 1978; Warburg, Sjo, Fledelius, & Pedersen, 1993). On the other hand, An et al. retrospectively analyzed 32 affected cases and reported that two genes expressed in the nervous system, *SOX12* and *NRSN2*, are candidate genes involved in developmental deficits (An et al., 2013). However, an animal model study identified the transcription factors *Sox4* and *Sox11* as important regulators of diverse developmental processes in various organ systems, including the nervous system, heart, lung, spleen, and pancreas, as well as β‐cell development (Hoser et al., 2008). Although Sox4, Sox11, and Sox12 are jointly referred to as SoxC proteins, *Sox12* is a weaker transactivator than the others (Hoser et al., 2008). *Sox12*‐deficient mice of both sexes were reported to exhibit normal fertility with no gross phenotypic abnormalities, suggesting that *Sox4* and *Sox11* compensate for the loss of function of *Sox12* during mouse organogenesis (Hoser et al., 2008). This discrepant effect of a defect in *Sox12* on embryonic development may be related to the presence or absence of functionally compensatory genes (Hoser et al., 2008), as well as to differences in the genetic backgrounds of humans and mice. Further studies are warranted to elucidate the precise roles of *SOX12* and *NRSN2* in developmental processes.

The patient in this case was diagnosed at a younger age than those in previously reported cases. Accordingly, we were unable to assess her developmental function. However, she harbored a large‐scale genetic defect comprising a microdeletion of up to 2.01 Mb in 20p13. This microdeletion, which was confirmed by oligonucleotide aCGH analysis, showed that the involved region contained *TBC1D20, SOX12*, and *NRSN2*. These findings suggest that a long‐term follow‐up of the subject's cognitive ability is needed to provide a sufficiently early intervention. Even though our review of previously reported patients failed to clarify a specific genotype–phenotype association in 20p13 microdeletion syndrome, we still demonstrated that the most common presentation was motor and language delay, followed by abnormal digits, mental retardation, large fontanelle, EEG abnormalities, and seizure. Our findings suggest that aCGH should be considered as a screening tool for any patients with the above presentations.

## ETHICAL COMPLIANCE

Signed informed consent was obtained from the patient's parents in accordance with Institutional Review Board of Tri‐Service General Hospital. The ethics committee approved this study.

## CONFLICT OF INTEREST

The authors declare no conflict of interest.

## References

[mgg3739-bib-0001] An, Y. , Amr, S. S. , Torres, A. , Weissman, L. , Raffalli, P. , Cox, G. , … Shen, Y. (2013). SOX12 and NRSN2 are candidate genes for 20p13 subtelomeric deletions associated with developmental delay. American Journal of Medical Genetics. Part B, Neuropsychiatric Genetics, 162B(8), 832–840. 10.1002/ajmg.b.32187 24019301

[mgg3739-bib-0002] Baker, E. , Hinton, L. , Callen, D. F. , Altree, M. , Dobbie, A. , Eyre, H. J. , … Haan, E. (2002). Study of 250 children with idiopathic mental retardation reveals nine cryptic and diverse subtelomeric chromosome anomalies. American Journal of Medical Genetics, 107(4), 285–293. 10.1002/ajmg.10159 11840484

[mgg3739-bib-0003] Hoser, M. , Potzner, M. R. , Koch, J. M. , Bosl, M. R. , Wegner, M. , & Sock, E. (2008). Sox12 deletion in the mouse reveals nonreciprocal redundancy with the related Sox4 and Sox11 transcription factors. Molecular and Cellular Biology, 28(15), 10.1128/MCB.00338-08 PMC2493363 18505825

[mgg3739-bib-0004] Jezela‐Stanek, A. , Kucharczyk, M. , Pelc, M. , Gutkowska, A. , & Krajewska‐Walasek, M. (2013). 1.15 Mb microdeletion in chromosome band 20p13 associated with moderate developmental delay‐additional case and data's review. American Journal of Medical Genetics. Part A, 161A(1), 172–178. 10.1002/ajmg.a.35654 23165892

[mgg3739-bib-0005] Leclercq, S. , Maincent, K. , Baverel, F. , Tessier, D. L. , Letourneur, F. , Lebbar, A. , & Dupont, J. M. (2009). Molecular cytogenetic characterization of the first reported case of inv dup del 20p compatible with a U‐type exchange model. American Journal of Medical Genetics. Part A, 149A(3), 437–445. 10.1002/ajmg.a.32640 19206177

[mgg3739-bib-0006] Liegel, R. P. , Handley, M. T. , Ronchetti, A. , Brown, S. , Langemeyer, L. , Linford, A. , … Sidjanin, D. J. (2013). Loss‐of‐function mutations in TBC1D20 cause cataracts and male infertility in blind sterile mice and Warburg micro syndrome in humans. American Journal of Human Genetics, 93(6), 10.1016/j.ajhg.2013.10.011 PMC3852926 24239381

[mgg3739-bib-0007] Lupski, J. R. , & Stankiewicz, P. (2005). Genomic disorders: Molecular mechanisms for rearrangements and conveyed phenotypes. PLoS Genetics, 1(6), 10.1371/journal.pgen.0010049 PMC1352149 16444292

[mgg3739-bib-0008] Martsolf, J. T. , Hunter, A. G. W. , Haworth, J. C. , & Herrmann, J. (1978). Severe mental retardation, cataracts, short stature, and primary hypogonadism in two brothers. American Journal of Medical Genetics, 1(3), 291–299. 10.1002/ajmg.1320010305 677168

[mgg3739-bib-0009] McGill, A. K. , Pastore, M. T. , Herman, G. E. , Alliman, S. , Rosenfeld, J. A. , & Weaver, D. D. (2010). A tale of two deletions: A report of two novel 20p13 –> pter deletions. American Journal of Medical Genetics. Part A, 152A(4), 10.1002/ajmg.a.33339 20358616

[mgg3739-bib-0010] Moutton, S. , Rooryck, C. , Toutain, J. , Cailley, D. , Bouron, J. , Villega, F. , … Goizet, C. (2012). Dysmorphic features in subtelomeric 20p13 deletion excluding JAG1: A recognizable microdeletion phenotype? European Journal of Medical Genetics, 55(2), 151–155. 10.1016/j.ejmg.2011.12.009 22274139

[mgg3739-bib-0011] Saleh, M. , Kamath, B. M. , & Chitayat, D. (2016). Alagille syndrome: Clinical perspectives. The Application of Clinical Genetics, 9, 75–82. 10.2147/TACG.S86420 27418850 PMC4935120

[mgg3739-bib-0012] Sebat, J. , Lakshmi, B. , Malhotra, D. , Troge, J. , Lese‐Martin, C. , Walsh, T. , … Wigler, M. (2007). Strong association of de novo copy number mutations with autism. Science, 316(5823), 445–449. 10.1126/science.1138659 17363630 PMC2993504

[mgg3739-bib-0013] Trachoo, O. , Assanatham, M. , Jinawath, N. , & Nongnuch, A. (2013). Chromosome 20p inverted duplication deletion identified in a Thai female adult with mental retardation, obesity, chronic kidney disease and characteristic facial features. European Journal of Medical Genetics, 56(6), 319–324. 10.1016/j.ejmg.2013.03.011 23542666

[mgg3739-bib-0014] Warburg, M. , Sjo, O. , Fledelius, H. C. , & Pedersen, S. A. (1993). Autosomal recessive microcephaly, microcornea, congenital cataract, mental retardation, optic atrophy, and hypogenitalism. Micro syndrome. American Journal of Diseases of Children, 147(12), 10.1001/archpedi.1993.02160360051017 8249951

